# Potentiation of the altered immune microenvironment following RF ablation of murine distant tumors with CTLA-4 immunotherapy

**DOI:** 10.1186/s41747-026-00765-4

**Published:** 2026-06-23

**Authors:** Kun Zhao, Yuhan Shen, Wei Yang, Hao Wu, Bing Wang, Wenbo Wang, Wei Wu, Kun Yan, S. Nahum Goldberg

**Affiliations:** 1 Key Laboratory of Carcinogenesis and Translational Research (Ministry of Education/Beijing), Department of Ultrasound, Peking University Cancer Hospital & Institute, Beijing, China; 2 Division of Image-Guided Therapy, Department of Radiology, Hadassah Hebrew University Medical Center, Jerusalem, Israel

**Keywords:** Immunotherapy, Mice, Radiofrequency ablation, RNA-seq, Tumor microenvironment

## Abstract

**Objective:**

Previous studies on the immune function after radiofrequency ablation (RFA) mainly focused on the immune microenvironment in residual tumors. Thus, we investigate the impact of complete RFA (cRFA) and incomplete RFA (iRFA) on the immune microenvironment of distant tumors, determining whether combination with immunotherapy can improve outcomes.

**Materials and materials:**

Using a bilateral subcutaneous CT26 murine model, one tumor underwent cRFA or iRFA while the contralateral tumor served as a distant site. Immune profiling by ribonucleic acid (RNA) sequencing, flow cytometry, and immunohistochemistry was performed at days 3 and 9 post-RFA (6 mice per group). Based on transcriptomic findings, anti-CTLA-4 therapy was evaluated following cRFA. Tumor growth and survival were additionally assessed (8 mice per group).

**Results:**

Infiltration of CD8^+^ T-cells post-RFA increased at day 3 compared with controls (*p* = 0.049), while decreasing at day 9 after cRFA. Additionally, the ratio between pro-inflammatory and anti-inflammatory macrophages (M1/M2) decreased at day 9 (*p* = 0.034), suggesting an immunosuppressive microenvironment. RNA-seq demonstrated that CTLA-4 significantly upregulated 9 days after cRFA. Combined cRFA+anti-CTLA-4 increased distant tumor CD8^+^ T-cells, and distant tumor growth in this group was significantly decreased *versus* cRFA alone (*p* = 0.040).

**Conclusion:**

Complete RFA is associated with a delayed immunosuppressive shift in distant tumors. Targeting CTLA-4 following ablation may partially mitigate this effect and enhance systemic tumor control.

**Relevance statement:**

The combination of anti-CTLA-4 targeted therapy and complete radiofrequency ablation demonstrated a synergistic anti-tumor immunotherapy effect, which provided a potentially better therapeutic strategy for preventing tumor recurrence after radiofrequency ablation.

**Key Points:**

- The RNA-seq results demonstrated that CTLA-4 was upregulated in the distant tumor on 9 days after complete radiofrequency ablation.
- Anti-tumor immune response initially associated with radiofrequency ablation was relatively transient, while an immunosuppressive microenvironment was formed in the distant tumor in 9 days.
- Anti-CTLA-4 therapy after complete ablation significantly delayed distant tumor growth and promoted an anti-tumor immune microenvironment.

**Graphical Abstract:**

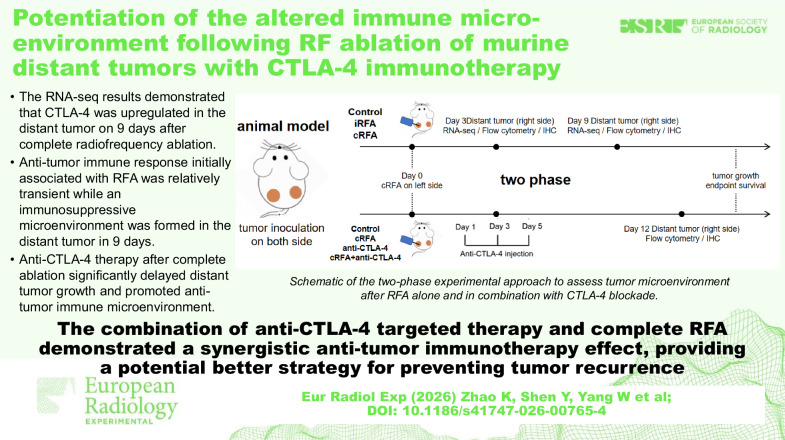

## Background

Radiofrequency ablation (RFA), as a minimally invasive treatment, is commonly used to treat focal primary hepatocellular carcinoma and oligo-metastatic liver cancer, such as colorectal cancer and breast cancer [1–3]. The clinical efficacy of RFA has been shown to approximate that of surgical resection for small liver tumors [4, 5]. In addition to direct tumoricidal effects of heat-induced coagulation, previous research has indicated that RFA significantly facilitates the activation of adaptive anti-tumor immunity by destroying tumors and releasing a large amount of tumor antigens [6–9]. Nevertheless, it has been observed that the tumor immune response after RFA is most often insufficient to suppress tumor recurrence and progression, especially recurrence and progression of distal tumors after RFA [10].

Current research on the tumor immune microenvironment post-RFA has mainly focused on the impact of incomplete radiofrequency ablation (iRFA) on residual tumors, while there is limited knowledge about the changes in the tumor immune microenvironment after complete radiofrequency ablation (cRFA). Moreover, although reports of providing immune-oncologic therapy in conjunction with tumor ablation in clinical practice are increasing [11–13], the data supporting precise mechanistic roles for immunotherapy combined with RFA are still insufficient. Yet, a better understanding of the changes in the immune microenvironment after RFA, and the development of more precise combined treatment strategies based on well-studied molecular rationales, is crucial for improving the survival of patients receiving RFA.

Incomplete RFA leaves residual viable tumor cells exposed to sublethal heat stress, which can promote a pro-inflammatory and immunosuppressive tumor microenvironment [14–16]. Although complete tumor ablation is the most common clinical goal, inevitably, there are cases where micrometastases and small non-visualized hepatocellular carcinomas are present [17, 18]. However, an immune-suppressed microenvironment can still develop, and we postulate that it is a key driver for tumor recurrence [19–21]. Accordingly, the primary objectives of this study are to investigate the effects of cRFA and iRFA on the immune microenvironment of distant tumors and, second, to provide supporting evidence for the hypothesis of CTLA-4 checkpoint blockade inducing a delayed immunosuppressive shift after cRFA.

## Methods

### Experimental overview

**Fig. 1:**
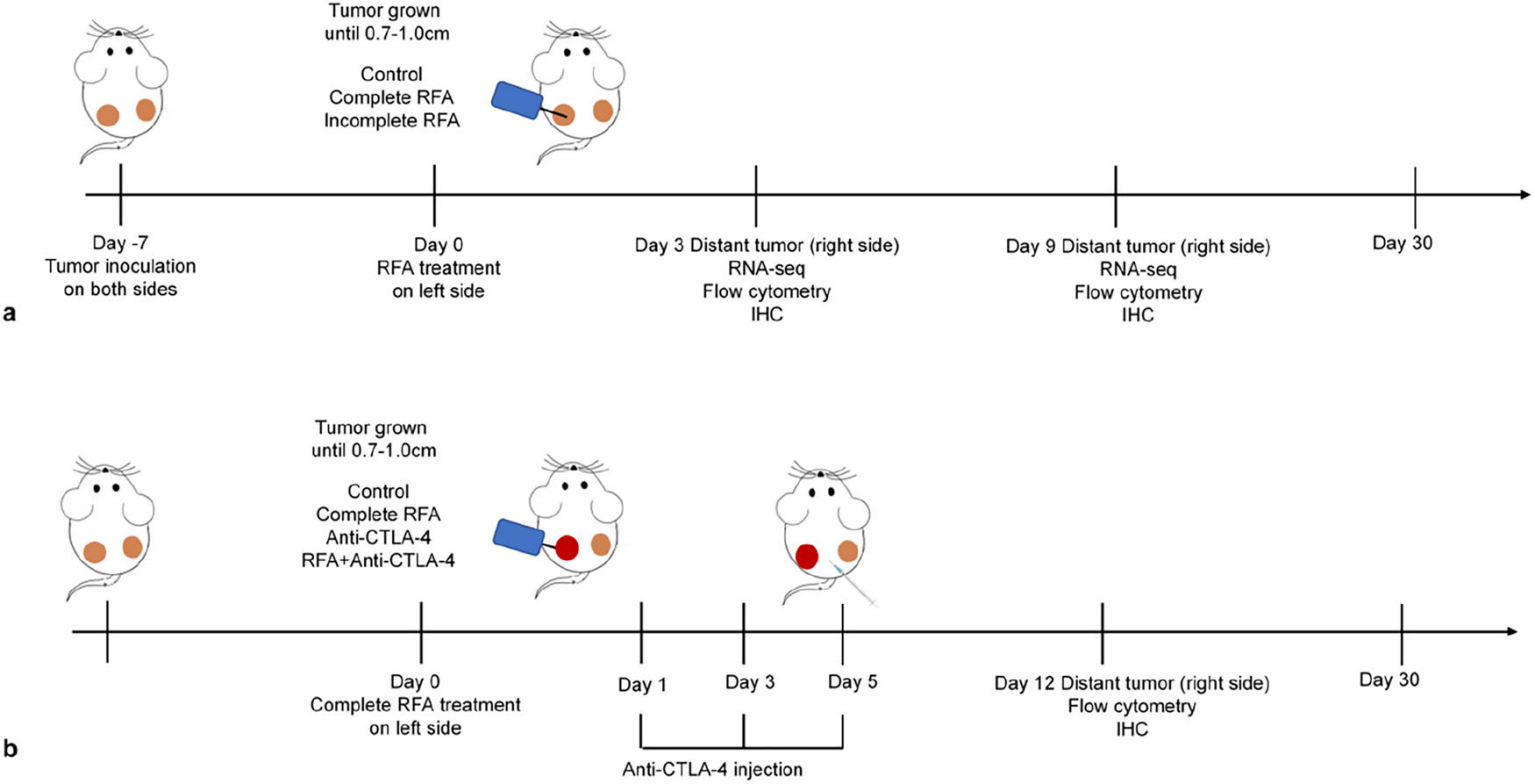
Illustration of the study design on the immune microenvironment of distant tumors following radiofrequency ablation (RFA). **a** The first phase explored the immune effects of complete RFA (cRFA) and incomplete RFA (iRFA) of a treated index tumor on a contralateral untreated tumor. Flow cytometry and immunohistochemical (IHC) were used for immune microenvironment analyses. **b** The second phase investigated the effect of complete RFA combined with anti-CTLA-4 antibody in this setting

The experimental animal protocol was approved by the Institutional Animal Care and Use Committee (Peking University Cancer Hospital) with approval number EAEC-2024-32. Our study was divided into two phases (Fig. 1). The first phase explored the effects of cRFA *versus* iRFA on the immune microenvironment of distant tumors, including RNA-seq, flow cytometry, and immunohistochemistry to analyze the immune microenvironment at day 3 (see early cellular activation and infiltration) and day 9 (to see more pronounced cellular changes) after RFA [22]. Based upon RNA-seq results, the second phase investigated the efficacy of cRFA combined with anti-cytotoxic-T-lymphocyte-associated antigen 4 (anti-CTLA-4) antibody on the distant tumors. Blinding was used for each step of the experimental process.

The primary endpoint was the inhibition of tumor growth, quantified as the difference in mean tumor volume between groups at day 12 after treatment initiation. Secondary endpoints were assessed to provide mechanistic insight, including transcriptomic analyses, immune cell infiltration, and endpoint survival. Transcriptomic analyses included gene expression profiles of distant tumors on days 3 and 9 after treatment, assessed by RNA-seq. Immune cell infiltration was assessed by measuring the frequency and phenotype of tumor-infiltrating CD8^+^ T-cells, Treg cells, and tumor-associated macrophages (TAMs), including the M1 phenotype (marked by CD80) and the M2 phenotype (marked by CD206) on days 3 and 9, by flow cytometry and IHC staining.

The survival endpoint was defined as a tumor volume of 2,000 mm^3^ or the follow-up period of 30 days, whichever reached first. The animals were sacrificed at the endpoint time.

### Animal model

A model of bilateral subcutaneous CT26 tumor-bearing mice was established. RFA was performed on one target tumor. The contralateral tumor served as the distant tumor.

All *in vivo* experiments were performed in female Balb/c mice weighing 20 ± 2 g (mean ± standard deviation) at 5–6 weeks (Beijing Huafukang Medical Experimental Animal Center). A total of 172 animals were used in the study, with 6–8 animals per group in the first and second experimental phases. All animals were fed with standard food and water under constant temperature and humidity. A 12-h light-dark cycle was implemented in the animal housing. For all experiments, anesthesia was induced by injecting pentobarbital sodium intraperitoneally. Animals were sacrificed with an overdose of carbon dioxide in a CO_2_ chamber. Mice were randomly co-housed in standard cages to minimize potential litter effects. After 1 week of acclimatization, all mice were subcutaneously inoculated with CT26 cells (at a density of 1 × 10^7^/mL) on the right flank. Tumor growth was monitored every 2 days. Previous experiments had confirmed that tumor size with a diameter ranging from 7 to 15 mm was suitable for RFA [23–25]. Therefore, tumors with a diameter of 7 to 10 mm were included in our study. The experimental randomization was performed 1 day before RFA. Mice were randomly assigned to each group based on their unique ear tag numbers, using a block randomization list generated by a random number algorithm in Excel. The group allocation sequence was prepared by a researcher not involved in the subsequent experiments, which was concealed from the investigators responsible for treatment administration and outcome assessments.

### Radiofrequency ablation

A 480-kHz radiofrequency generator (SINOSURGICAL Medical Technology Co., Ltd.) and the 0.7-cm tip of a 17-gauge monopolar electrode were used. To achieve the circuit required for RFA, the animals were placed on a conventional electrode pad. The animal’s back was shaved, and contact gel was applied to facilitate conductivity. In the cRFA group, the radiofrequency electrode needle was placed at the center of the tumor to perform center RFA. In the iRFA group, the radiofrequency electrode needle was placed on one side of the tumor to create an eccentric RFA coagulation zone. For both conditions, the RFA generator was set to achieve a tip temperature of 70 ± 2 °C (mean ± standard deviation) with energy applied for 5 min. The animals were excluded if the tumor was not totally necrotic after cRFA or if the animal died prematurely.

### Anti-CTLA-4 antibody injection

Based on prior relevant experiments [26, 27], 200 μg of anti-CTLA-4 antibody (clone 9D9 monoclonal antibody, #BP0164; BioxCell) was dissolved in 0.2 mL of phosphate-buffered saline, resulting in a drug concentration of approximately 10 mg/kg. Intraperitoneal injection was chosen as the administration method in the study. The anti-CTLA-4 antibody was administered on days 1, 3, and 5 after RFA (0.2 mL for each mouse).

### Tumor growth rates and endpoint survival

In the first phase, mice bearing CT26 tumors were randomly allocated into three equal groups (*n* = 8 per group) as follows: (1) control (no treatment); (2) iRFA; and (3) cRFA. In the second phase, mice were randomly divided into four groups (*n* = 8 per group): (1) control (no treatment); (2) cRFA alone; (3) anti-CTLA-4 antibody alone; and (4) cRFA + anti-CTLA-4 antibody group. During the follow-up period of 30 days, the diameter of the tumor and the body weight of each mouse were measured every 2 days. The tumor volume was calculated as D × d^2^/2, where D was the maximum diameter and d is the minimum diameter.

### Evaluation of the immune microenvironment and pathological changes

In the first phase, the 3-day period after RFA was defined as the early adaptive activation. The 9-day period after RFA was defined as the late immune response [28, 29]. Mice with CT26 tumors were randomly divided into three groups (*n* = 6 per group): (1) control group (no treatment); (2) iRFA group; and (3) cRFA group. After RFA, distal tumor tissues were harvested for RNA-seq, flow cytometry, and immunohistochemistry to analyze the effects on the tumor immune microenvironment. The details of RNA-seq, flow cytometry, and immunohistochemistry are described in the Supplementary material.

In the second phase, the mice with tumors were randomly divided into four groups (*n* = 6 per group): (1) control (no treatment), (2) cRFA, (3) anti-CTLA-4 antibody, and (4) cRFA + anti-CTLA-4 antibody. Given relevant differences in growth by day 12 post-RFA, animals were sacrificed, and distal tumor tissues were harvested and sectioned. Flow cytometry and immunohistochemistry analyzed the changes in the immune microenvironment.

### Statistical analysis

All continuous variables were presented as mean ± standard deviation and 95% confidence interval (CI) where possible. The Kruskal–Wallis test was used to evaluate the difference in quantitative comparison of body weight, flow cytometric assay, and cell expression in different groups. Repeated measures ANOVA was used to compare the differences in tumor volume at different time points during follow-up. Survival analysis of the endpoint was performed using the Kaplan–Meier method. The Log-rank test was used for comparison. Given that more than two groups were involved in this survival study, the Log-rank test with Bonferroni correction was employed to specifically compare two targeted groups only when the overall *p* value was less than 0.05. SPSS 21.0 statistical analysis software was used to analyze the data. The *p*-values < 0.05 were considered statistically significant. A tumor signaling-related protein network was constructed by the Search Tool for the Retrieval of Interacting Genes/Proteins (STRING version 12.0; https://string-db.org/) with a 0.7 high confidence score. Genes were clustered by the Markov Cluster algorithm with an inflation parameter set as 2. The Metascape database [https://metascape.org] and Reactome Pathway Database [https://reactome.org] were used for functional enrichment analysis based upon the differential gene list.

## Results

### Immune microenvironment of distant tumor 3 days after RFA

#### Cellular analysis

**Fig. 2:**
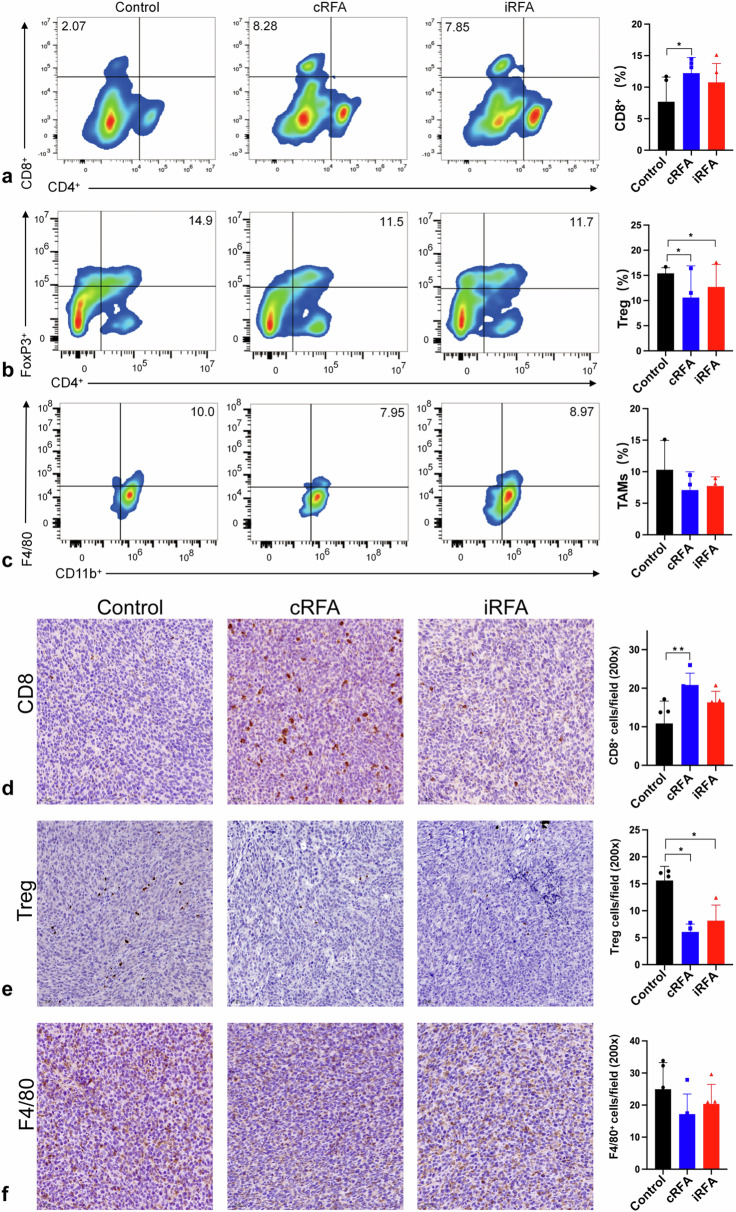
Induction of immune-related cells infiltration and IHC results of distant tumor 3 days after RFA. Figure displays and quantifies: **a**, **d** the percentage and expression of CD8^+^ T cells; **b**, **e** the percentage and expression of Tregs (regulated-T cells); **c**, **f** the percentage and expression of tumor-associated macrophages (TAMs). Overall, this shows an increase in CD8^+^ T cell infiltration and a reduction of Tregs, suggesting the activation of immune response in the short term after both cRFA and iRFA in distant tumors

Flow cytometric results showed that the percentage of CD8^+^T cells infiltration was increased in the cRFA group (11.16 ± 2.77%, 95% CI: 8.25–14.07%) and iRFA group (10.43 ± 4.05%, 95% CI: 6.18–14.68%), compared with the control group (8.29 ± 5.40%, 95% CI: 2.62–13.96%, *p* = 0.049, *p* = 0.083) (Fig. 2a). The infiltration percentage of Treg cells was decreased significantly in cRFA group (10.59 ± 6.31%, 95% CI: 3.97–17.21%), and in iRFA group (12.71 ± 4.48%, 95% CI: 8.01–17.41%) compared to control group (15.40 ± 1.14%, 95% CI: 14.20–16.60%, *p* = 0.028, *p* = 0.047) (Fig. 2b). Immunohistochemical results showed that on 3 days after RFA, the CD8^+^ T-cell expression was elevated in both the cRFA group (20.85 ± 2.72%, 95% CI: 18.00–23.71%) and the iRFA group (16.32 ± 2.58%, 95% CI: 13.61–19.03%) compared to the control group (10.87 ± 5.17%, 95% CI: 5.44–16.30%, *p* = 0.009, *p* = 0.251) (Fig. 2d). The expression of Treg cells was decreased significantly in cRFA group (6.08 ± 1.26%, 95% CI: 4.76–7.40%) and iRFA group (8.17 ± 2.50%, 95% CI: 5.55–10.79%) compared to control group (15.61 ± 2.27%, 95% CI: 13.23–17.99%, *p* = 0.021, *p* = 0.043) (Fig. 2e). However, the expression of TAMs was not changed significantly in cRFA group (17.18 ± 0.56%, 95% CI: 16.59–17.77%) and iRFA group (20.35 ± 5.45%, 95% CI: 14.63–26.07%) compared to control group (24.95 ± 7.42%, 95% CI: 17.16–32.74%, *p* = 0.076, *p* = 0.347) (Fig. 2f).

#### Molecular pathways

**Fig. 3:**
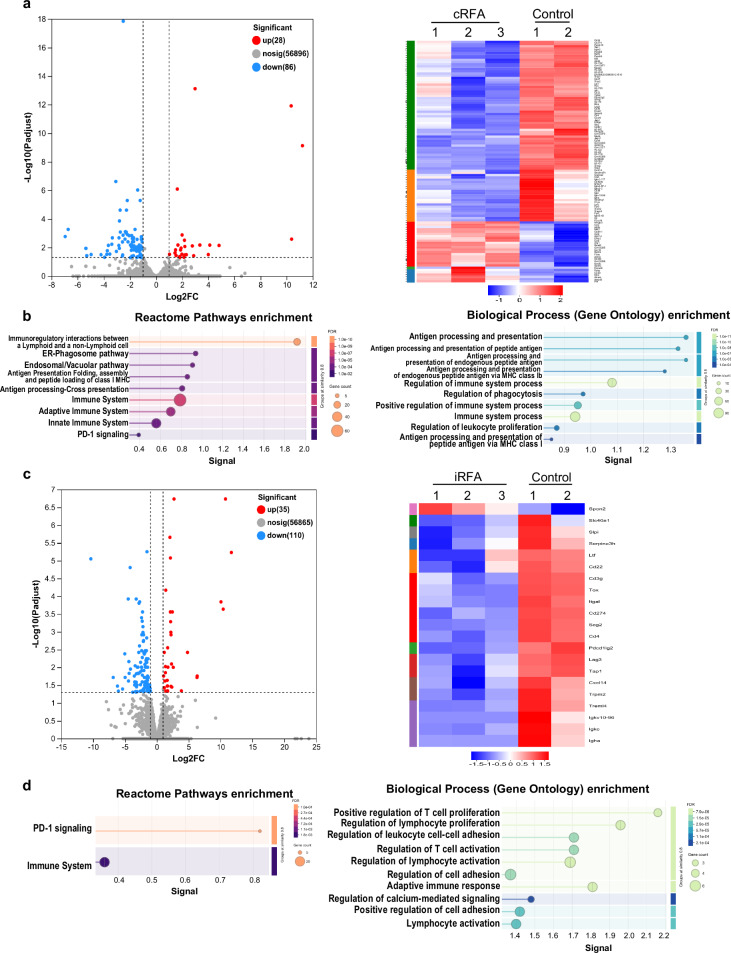
Genes associated with the immune system process altered 3 days after RFA. **a** Volcano plot and heatmap of mean fold-changes of genes after cRFA compared with the control group. **b** Reactome pathway and GOBP analysis of genes after cRFA compared with the control group. **c** Volcano plot and heatmap of mean fold-changes of genes after iRFA compared with the control group. **d** Reactome pathway analysis of genes after cRFA compared with the control group and the GOBP pathway analysis of the genes in cluster 1 (*i.e*., “PD-1 signaling”) demonstrate downregulated immune functions at 72 h after iRFA

The results of RNA-seq analysis at day 3 after RFA showed that there were 28 upregulated genes and 86 downregulated genes in distant tumors in the cRFA group compared with the control group (Fig. 3a). Reactome analysis revealed a total of 364 upregulated genes involved in the immune system process. To identify the signaling of up genes after cRFA, a protein–protein interaction network of distant tumors and immune-related signaling was constructed (Fig. S3a). “Gene ontology biological process” (GOBP) and Reactome pathway analysis demonstrated that the significant immune-related signaling pathways were mainly associated with the regulation of antigen processing and presentation (Fig. 3b). The genes in the top two Markov cluster (MCL) clusters were described as concerning PD-1 signaling and immunoregulatory interactions (Fig. S3b). Compared with the control group, there were 35 upregulated genes and 110 downregulated genes in the iRFA group (Fig. 3c). Of them, 96 downregulated genes were found to be related to the immune system and PD-1 signaling based on Reactome pathway analysis (Fig. 3d). Among the clusters, cluster 1, denoted by PD-1 signaling as the most significant group in relation with T-cell regulation according to GOBP and KEGG analysis (Figs. 3d, S3c).

These data suggest activation of the immune response in the short term after RFA, and the expression levels of the genes associated with pathways related to the immune system were predominantly upregulated after cRFA while downregulated after iRFA.

### Immune microenvironment of distant tumor 9 days after RFA

#### Cellular analysis

**Fig. 4:**
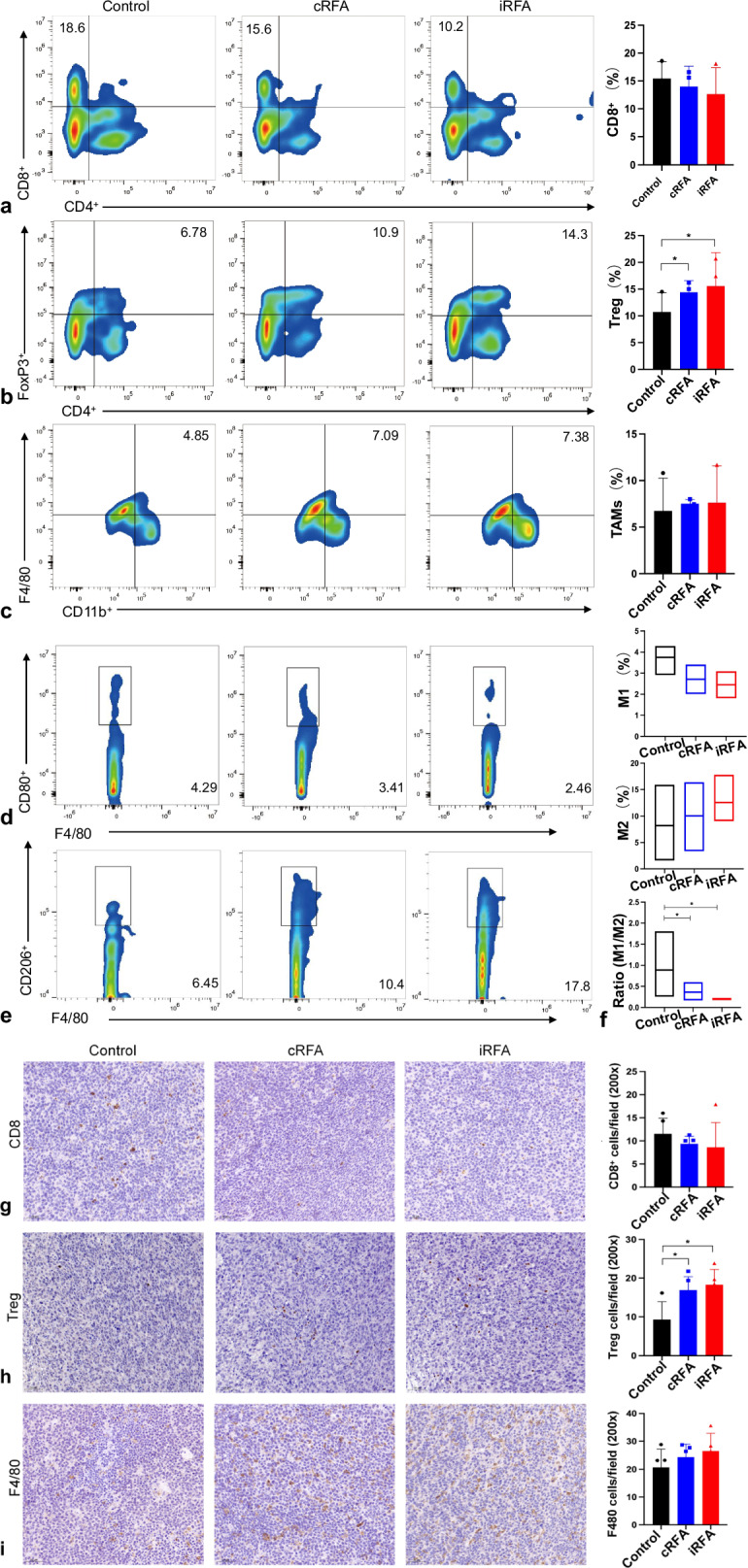
Induction of immune-related cells infiltration and IHC results of distant tumor at 9 days after RFA. Figure displays and quantifies: percentage and expression of CD8^+^T cells (**a**, **g**); percentage and expression of Tregs (**b**, **h**); percentage and expression of TAMs (**c**, **i**); (**d**) M1-type macrophages infiltration ratio; (**e**) M2-type macrophages infiltration ratio; and (**f**) M1/M2 ratio

Flow cytometry results showed that percentage of CD8^+^ T cells infiltration was similar in the cRFA group (14.01 ± 2.98%, 95% CI: 10.88–17.14%) and in the iRFA group (12.66 ± 3.85%, 95% CI: 8.62–16.70%) compared to the control group (15.43 ± 2.46%, 95% CI: 12.85–17.99%, *p* = 0.827, *p* = 0.275) (Fig. 4a). The percentage of the immunosuppressive Treg cells infiltration was significantly increased in both the cRFA group (14.40 ± 2.16%, 95% CI: 12.13–16.67%) and the iRFA group (15.57 ± 6.24%, 95% CI: 9.03–22.11%) compared to the control group (10.72 ± 3.58%, 95% CI: 7.00–14.44%, *p* = 0.020, *p* = 0.028) (Fig. 4b).

For quantitative analysis of tumor-associated macrophages, we observed a similar decrease in M1-type tumor-associated macrophages in both the cRFA group (2.71 ± 0.57%, 95% CI: 2.11–3.31%) and the iRFA group (2.45 ± 0.52%, 95% CI: 1.90–3.00%) compared to the control group (3.75 ± 0.60%, 95% CI: 3.12–4.38%, *p* = 0.127, *p* = 0.127) (Fig. 4d). Contemporaneously, M2-type tumor-associated macrophages were significantly increased in the cRFA group (10.05 ± 5.33%, 95% CI: 4.46–15.64%) and the iRFA group (12.59 ± 3.76%, 95% CI: 8.66–16.52%) (Fig. 4e). The M1/M2 ratios were significantly lower in both the cRFA group (0.36 ± 0.18) and the iRFA group (0.20 ± 0.02) compared with the control group (0.89 ± 0.66, *p* = 0.034 and *p* = 0.022, respectively) (Fig. 4f).

The immunohistochemical results were similar to the flow cytometry results. At day 9 after RFA, the expression of immunosuppressive Treg cells was significantly increased in both the cRFA (16.95 ± 3.06%, 95% CI: 13.74–20.16%) and the iRFA (18.28 ± 3.56%, 95% CI: 14.76–21.80%) groups compared with the control group (9.34 ± 3.98%, 95% CI: 5.16–13.52%, *p* = 0.036, *p* = 0.027) (Fig. 4h).

#### Molecular pathways

**Fig. 5:**
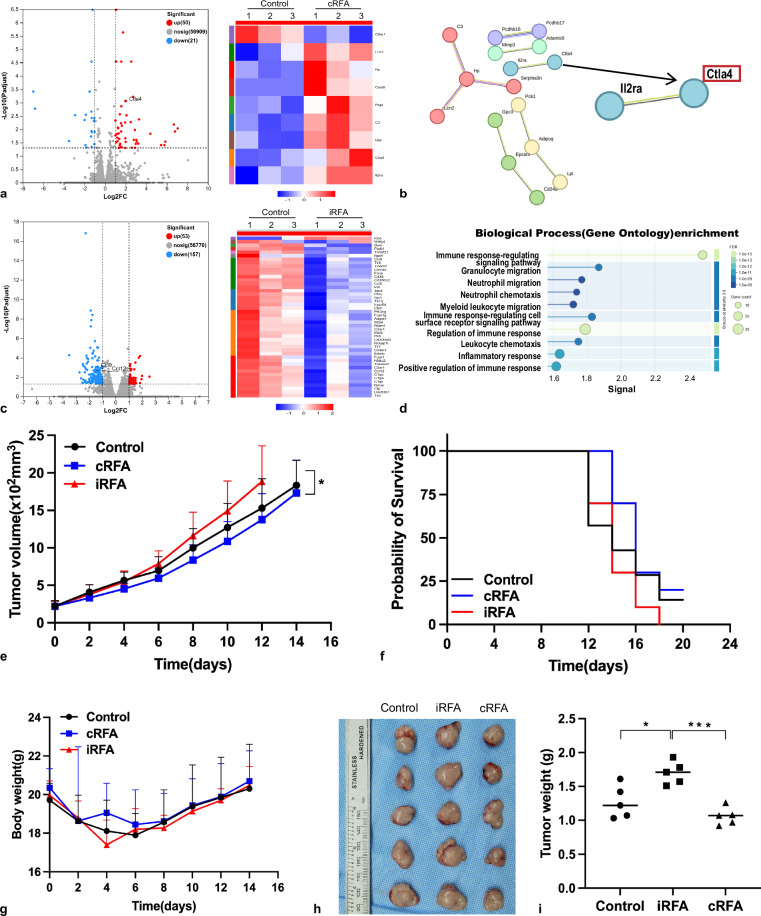
Comparison of regulated genes and tumor growth of distant tumor at 9 days after cRFA and iRFA. **a** The volcano plot and heatmap of mean fold-change after cRFA compared with the control group. **b** The protein–protein interaction network demonstrates that CTLA-4 is upregulated at 9 days after cRFA. **c** Volcano plot, heatmap of mean fold-change after iRFA compared with the control group. **d** GOBP enrichment analysis of genes demonstrates multiple downregulated immune functions after iRFA. The growth curve (**e**), the survival curve (**f**), the weight curve (**g**), the tumor weight picture (**h**), and quantitative analysis of tumor weight (**i**) show that iRFA promotes tumor progression in the distant tumor

RNA-seq analysis of distant tumors at 9 days after RFA demonstrated 50 genes upregulated and 21 genes downregulated in the cRFA group compared to the control group (Fig. 5a). The protein–protein interaction network and enrichment analysis showed that CTLA-4 was upregulated in the cRFA group compared to the control group, which is associated with abnormal regulatory T cells (Figs. 5b, S4a, b). For the iRFA group compared to control group, 153 genes related to immune system process were screened in GO analysis, among which CCL9 and CCL12, related chemokines that promote the recruitment of bone marrow cells, were downregulated (Fig. 5c). Similarly, there were clusters of downregulated genes potentially attributable to immune system and immune response proved by GO and Reactome enrichment analysis (Figs. 5d, S4c).

These data indicate that the formation of an immunosuppressive microenvironment evolves by 9 days after RFA, and that these potentially negative effects of iRFA on the immune microenvironment of the distant tumor were stronger than cRFA.

### Tumor growth studies after complete or incomplete RFA

The tumor growth results showed that the distant tumor growth rate in the iRFA group (at day 12: 1,886.90.50 ± 472.89 mm^3^) was highest compared to the cRFA group (1,376.33 ± 348.20 mm^3^, *p* = 0.036) and control group (1,530.43 ± 396.67 mm^3^, *p* = 0.069) (Fig. 5e). For the endpoint survival, incomplete RFA group had shorter survival than the cRFA group (14 *versus* 17 days, *p* = 0.027) (Fig. 5f). The difference in the tumor growth rate between cRFA group and control group was not statistically significant (14 days, 1,777.10 ± 435.38 *versus* 1,857.80 ± 316.83 mm^3^, *p* = 0.787) (Fig. 5e, h, i). The endpoint survival time was also not different between the cRFA group and control groups (16 *versus* 14 days, *p* = 0.423) (Fig. 5f). There was no significant difference in body weight among the three groups (*p* = 0.565) (Fig. 5g).

### Remodeling of the immune microenvironment after combination with anti-CTLA-4

**Fig. 6:**
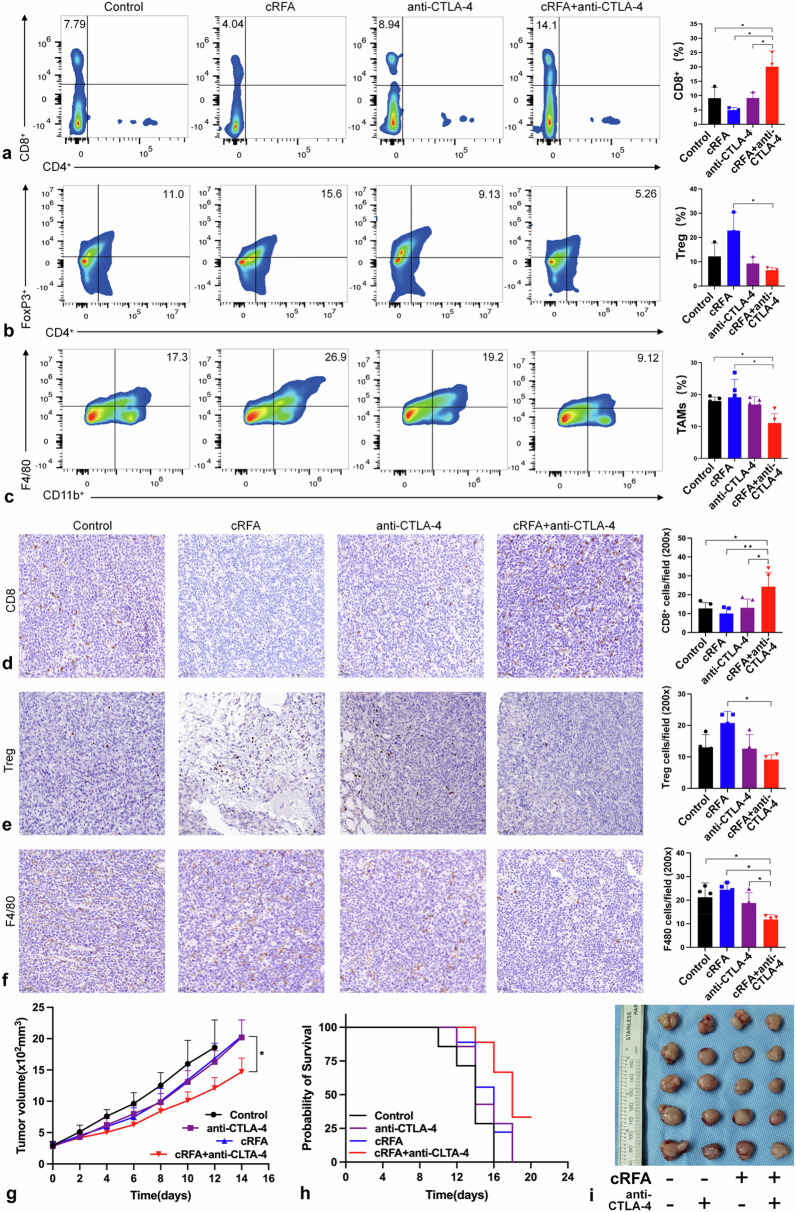
Induction of immune-related cellular infiltration of distant tumor combining cRFA with anti-CTLA. Figure displays and quantifies: **a**, **d** percentage and expression of CD8^+^ T cells; **b**, **e** percentage and expression of Tregs; **c**, **f** percentage and expression of TAMs. The growth curve (**g**), the survival curve (**h**), and the tumor picture (**i**) demonstrate that combined cRFA and anti-CTLA-4 treatment inhibited tumor progression and prolonged survival time

We assessed whether, combined with immunotherapy, anti-CTLA-4 could improve the tumor growth rate of the distant tumor and animal survival time after cRFA. The combined treatment group was found to exhibit a significant increase in the percentage of CD8^+^ T cells infiltration (20.05 ± 5.55%, 95% CI: 15.41–24.69%) compared with that in the control group (9.12 ± 3.70%, 95% CI: 6.03–12.21%, *p* = 0.049), cRFA group (5.97 ± 2.02%, 95% CI: 4.28–7.66%, *p* = 0.040) or anti-CTLA-4 group (9.17 ± 2.03%, 95% CI: 7.47–10.87%, *p* = 0.045) (Fig. 6a). The percentage of Treg cells infiltration in the combination treatment group was significantly reduced compared with cRFA group 6.48 ± 1.14%, 95% CI: 5.53–7.43% *versus* (22.83 ± 7.46, 95% CI: 16.59–29.07%, *p* = 0.028) (Fig. 6b). The infiltration ratio of TAMs was also lower in the combination group compared with control group (11.08 ± 2.95, 95% CI: 8.61–13.55% *versus* 18.00 ± 1.25%, 95% CI: 16.96–19.04%, *p* = 0.047) and cRFA group (11.08 ± 2.95 *versus* 19.14 ± 5.60%, 95% CI: 14.46–23.82%, *p* = 0.049) (Fig. 6c).

Similarly, the immunohistochemical results also showed that the expression of CD8^+^ T cells in the combined treatment group (24.27 ± 6.78%, 95% CI: 18.60–29.94%) was significantly higher compared to the control group (12.77 ± 2.82%, 95% CI: 10.41–15.13%, *p* = 0.016), the cRFA group (6.10 ± 2.50%, 95% CI: 4.01–8.19%, *p* = 0.009), and the anti-CTLA-4 group (9.07 ± 1.22%, 95% CI: 8.05–10.09%, *p* = 0.019) (Fig. 6d). Compared with the cRFA group, the expression of Treg cells in the combined treatment group was significantly decreased (20.77 ± 3.21 *versus* 9.15 ± 1.28%, *p* = 0.029) (Fig. 6e). The expression of TAMs in the combined treatment group was also decreased (11.81 ± 1.74 *versus* 21.26 ± 5.20%, *p* = 0.046, 11.81 ± 1.74 *versus* 24.44 ± 2.67%, *p* = 0.028, 11.81 ± 1.74 *versus* 18.81 ± 3.75%, *p* = 0.117) (Fig. 6f).

### Distant tumor growth is reduced after complete RFA combined with anti-CTLA-4

The distant tumor growth rate in combined RFA with anti-CTLA-4 (at day 14: 1,470.17 ± 220.65 mm^3^) was the slowest compared with that in the control group (1,860.43 ± 438.56 mm^3^, *p* = 0.002) and the cRFA group (2,040.72 ± 256.84 mm^3^, *p* = 0.033) and anti-CTLA-4 group (2,019.71 ± 279.75 mm^3^, *p* = 0.024) (Fig. 6g). For the endpoint survival, the survival of mice receiving combined RFA with anti-CTLA-4 (18 days) was significantly prolonged compared to the control (14 days, *p* = 0.002), cRFA (16 days, *p* = 0.022) and anti-CTLA-4 (14 days, *p* = 0.035) group (Fig. 6h). There was no significant difference in body weight between different treatment groups during follow-up (*p* = 0.126) (Fig. 6i).

## Discussion

Image-guided RFA has been widely used in the treatment of primary and metastatic focal liver tumors. In contrast to surgical resection, RFA induces coagulative necrosis of the local tumor, resulting in the release of large amounts of cellular debris *in situ*, which can serve as a source of tumor antigens, be taken up by antigen-presenting cells, and induce specific immune responses [30]. In previous studies on animal models and patients with liver tumors, it was observed that RFA could induce a specific T-cell immune response [6, 31]. RFA also induced activation of the liver distant from the ablation zone, altering immunoregulatory pathways [32]. However, subsequent studies had confirmed that the immune response induced by RFA was insufficient to prevent tumor recurrence after RFA, and the recurrence rate was higher than that of surgical resection, especially for large liver tumors [33, 34]. In this study, we investigated the effects of cRFA and iRFA on the immune microenvironment of distant tumors by establishing a model of bilateral subcutaneous tumor-bearing mice, providing data evidence for changes in the immune microenvironment after RFA and the combination of RFA with immunotherapy.

The study found that a favorable anti-tumor immune response initially formed after RFA was relatively transient. Indeed, by 9 days after RFA, an immunosuppressive microenvironment was formed in the distant tumor tissue. It was proven that iRFA was associated with a reduced survival duration relative to the control cohort. Moreover, there were more immune system-related genes present in the setting of iRFA compared to cRFA, and the microenvironment of the residual tumors varied more distinctly compared to which in the distant tumors. Our study is in concert with the negative immunologic and protumorigenic findings post- RFA seen in prior studies. For example, Shi et al [28] showed that at 8 days after cRFA in the CT26 tumor-bearing mouse model, the amount of functional infiltrating T cells was significantly reduced and the expression of PD-L1/PD-1 was upregulated, rapidly overcoming the immune response. By the same token, in a study on the recurrence of hepatocellular carcinoma after RFA in a chronic inflammatory mouse model, Rosenbloom et al [35] found that RFA and surgical resection induced an increase in tumor burden and a significant reduction in survival rate compared with the control group, with no significant difference between RFA and surgical resection.

In the comparison between the cRFA group and the iRFA group, we focused on the differential genes related to immune system regulation identified by RNA-seq enrichment analysis. At day 3 after RFA, no significantly differentially expressed genes were identified between the two groups. However, at day 9 after RFA, the results showed that CXCL13, a member of the chemokine family, was upregulated in the cRFA group. CXCL13 was first proposed as a marker of T-cell exhaustion [36], but later studies found that CXCL13 could promote the recruitment of CXCR5^+^ CD8^+^ T cells. In patients with high-grade serous ovarian cancer, increased expression of CXCL13 could shape immunocompetent cells and effectively prolong the survival time of patients [37]. However, in breast cancer research, it is generally believed that CXCL13-CXCR5 coexpression drives disease progression and metastasis. In the mouse model of breast cancer, the addition of CXCL13 inhibitors could promote cell apoptosis, resulting in the reduction of tumor volume and growth rate, and this effect may be related to the CXCR5/ERK signaling pathway [38, 39]. It could be seen that the role of the chemokine CXCL13 may vary in different tumor types, and the findings in this study provided a possible direction for subsequent comparative studies of cRFA and iRFA.

In this study, flow cytometry, immunohistochemistry, and growth curves were used for analysis. The results demonstrated that compared to the iRFA group, the growth trend of distal tumors in the cRFA group was relatively slower, and the percentage of CD8^+^ T cells infiltration was relatively higher, indicating that cRFA had a better therapeutic effect. However, the specific underlying mechanisms still require further exploration and discussion through subsequent experiments.

Our research has further demonstrated that cRFA was insufficient to enhance effective anti-tumor immunity and had no significant effect on reducing distant tumor metastasis. At present, much research on immune checkpoint inhibitors has focused on PD-L1/PD-1. Many studies have confirmed that RFA combined with anti-PD-1 antibody could inhibit tumor growth and prolong survival time [10, 40–42]. However, in this study, RNA-seq results at 9 days after RFA showed that CTLA-4 expression was significantly upregulated in the cRFA group, while PD-1 expression was not the most upregulated gene. CTLA-4 was an immune checkpoint molecule expressed by T cells, which was constitutively expressed in regulatory T cells. When bound to CD80 or CD86 on the surface of antigen-presenting cells, CTLA-4 acted as an immune checkpoint to downregulate the immune response [43, 44]. As another immune checkpoint molecule, T cells could be activated by the addition of immune checkpoint inhibitors [45].

Based on this, we established a bilateral tumor-bearing mouse model to verify that cRFA combined with anti-CTLA-4 antibodies regulates anti-tumor immune responses, significantly increasing the percentage of CD8^+^ T cells infiltration in distant tumor tissues and reducing the percentage of Treg cells infiltration. The distant tumor growth rate was significantly slowed, and survival time was significantly prolonged, which can help improve the therapeutic effect. This result confirmed that combination therapy was an effective treatment strategy that provided potential curative methods for patients and warrants further investigation in clinical trials. Similarly, Zhang et al [46] also reported that RFA combined with anti-CTLA-4 antibody increased the infiltration of CD4^+^ and CD8^+^ lymphocytes. After tumor restimulation, 75% of the mice in the combined group had obvious tumor rejection, which proved that the combined therapy could enhance the systemic anti-tumor immunity induced by RFA. In clinical applications, Duffy et al [47] found that Tremelimumab (an anti-CTLA-4 drug) combined with RFA was a potential new treatment for advanced hepatocellular carcinoma. Among the 19 evaluable patients, 5 achieved confirmed partial remission, CD8^+^ T cells increased significantly, and there may be an alternative reduction in hepatitis C viral load.

We note some limitations in our study. First, the conclusions were based on small sample size animal tumor models, which may not fully reflect the changes in human tumors. Second, the study was only experimentally validated in one tumor model. The observed modest differences in survival between the cRFA and control groups may be attributed to the context of inherent inter-animal variability and the biological relevance of the effect size. Although CT26 is a classic tumor immune research model, whether similar results could be obtained in other tumor models remains to be further explored. Finally, given the complexity of the immune microenvironment of tumors, future studies should more fully explore post-ablation effects on additional cellular populations and relevant mechanisms of their post-ablation response. While our study establishes the efficacy of combined CTLA-4 antibody with RFA in a subcutaneous model, translating these findings to the clinic requires addressing evaluation in orthotopic models and optimal post-RFA treatment protocols.

All in all, the first phase results showed that an immune-suppressed microenvironment was formed, and CTLA-4 was most significantly upregulated after cRFA at day 9. Second phase confirmed combination cRFA with anti-CTLA-4 significantly decreased tumor growth and increased endpoint survival, with the percentage of CD8^+^ T cells of distant tumors increased significantly. Also, we observed that the anti-tumor immune response induced in the short term could not significantly prevent the local tumor recurrence and the metastasis of distant tumors after RFA. The observed synergy between complete RFA and anti-CTLA-4 in this study provides a rationale for translational investigation into this combination regimen for solid cancer treatment.

## Supplementary information

**Additional File 1: Fig S1.** Study design flow chart CONSORT-style flowchart detailing animal allocation, experimental cohorts, and analysis pathways.**Fig S2.**Individual results from flow cytometry analysis show the populations of CD8⁺ T cells, Tregs, and TAMs on Day 3.**Fig S3.** Genes associated with immune system process were induced 3 days after RFA. (**a** The MCL clusters of genes after complete RFA compared with the control group. (**b**) The Reactome pathway analysis of up-regulated genes in the top 2 clusters showed that immune system and PD-1 signaling were involved. (**c**) The PPI network and the KEGG pathway analysis of the genes in cluster1 described as PD-1 signaling demonstrates down-regulated immune functions at 72 hours after iRFA.** Fig S4.**Genes associated with immune system process were induced 9 days after RFA. (**a**) The MCL clusters of genes at 9 days after complete RFA compared with the control group showed demonstrates that CTLA-4 was up-regulated. (**b**) MPO enrichment analysis of genes after complete RFA compared with the control group. (**c**) PPI plot and Reactome Pathways enrichment analysis of 60 genes down-regulated demonstrates multiple down regulated immune functions after incomplete RFA.** Fig S5.**Tumor growth trajectories with 95% confidence interval bands and individual per-mouse tumor growth of phase 1.

## Data Availability

The datasets generated and/or analyzed during the current study are not publicly available due to the policies and confidentiality agreements adhered to in our laboratory but are available from the corresponding author on reasonable request.

## Abbreviations

CI: Confidence interval
cRFA: Complete radiofrequency ablation
CTLA-4: Cytotoxic-T-lymphocyte-associated antigen 4
GOBP: Gene ontology biological process
iRFA: Incomplete radiofrequency ablation
RFA: Radiofrequency ablation
RNA-Seq: Ribonucleic acid sequencing
TAMs: Tumor-associated macrophages
