# Supplementary material for: Potentiation of the altered immune microenvironment following RF ablation of murine distant tumors with CTLA-4 immunotherapy
Source: Eur Radiol Exp. 2026 Jun 23;10:97. doi: 10.1186/s41747-026-00765-4 (PMC13291295; doi:10.1186/s41747-026-00765-4)
Supplement: Supplementary file 1 — Additional File 1: Fig S1. Study design flow chart CONSORT-style flowchart detailing animal allocation, experimental cohorts, and analysis pathways.Fig S2.Individual results from flow cytometry analysis show the populations of CD8⁺ T cells, Tregs, and TAMs on Day 3.Fig S3. Genes associated with immune system process were induced 3 days after RFA. (a The MCL clusters of genes after complete RFA compared with the control group. (b) The Reactome pathway analysis of up-regulated genes in the top 2 clusters showed that immune system and PD-1 signaling were involved. (c) The PPI network and the KEGG pathway analysis of the genes in cluster1 described as PD-1 signaling demonstrates down-regulated immune functions at 72 hours after iRFA. Fig S4.Genes associated with immune system process were induced 9 days after RFA. (a) The MCL clusters of genes at 9 days after complete RFA compared with the control group showed demonstrates that CTLA-4 was up-regulated. (b) MPO enrichment analysis of genes after complete RFA compared with the control group. (c) PPI plot and Reactome Pathways enrichment analysis of 60 genes down-regulated demonstrates multiple down regulated immune functions after incomplete RFA. Fig S5.Tumor growth trajectories with 95% confidence interval bands and individual per-mouse tumor growth of phase 1. [file 41747_2026_765_MOESM1_ESM.pdf]

Potentiation of the altered immune microenvironment following RF ablation of murine distant tumors with CTLA-4 immunotherapy

ELECTRONIC SUPPLEMENTARY MATERIAL

RNA sequencing

Total RNA was extracted from the tissue using TRIzol® Reagent according the manufacturer's instructions. Then RNA quality was determined by 5300 Bioanalyser (Agilent) and quantified using the ND-2000 (NanoDropTechnologies). Only high-quality RNA sample. (OD260/280= 1.8~2.2, OD260/230≥2.0, RIN≥ 6.5, 28S:18S≥ 1.0, > 1μg) was used to construct sequencing library.

Library construction and sequencing were performed according to the instructions of Illumina NovaSeq 6000 (San Diego, CA).

The raw paired end reads were trimmed and quality controlled by Fastp (Version 0.23.4). Then clean reads were separately aligned to reference genome with orientation mode using STAR (Version 2.7.1a) software.

RSEM (Version 1.3.3) was used to quantify gene abundances. Differential expression analysis was performed using the DESeq2 (Version 1.42.0). The mapped reads of each sample were assembled by StringTie (Version 2.2.1). Differential expression genes with |log2FC| ≧ 1 and FDR≤ 0.05 were considered to be significantly different expressed genes.

Flow cytometry

The tumor tissue was minced and homogenized, and then a collagenase digestion solution was added to prepare a single-cell suspension. Red blood cell lysis buffer was added to remove the red blood cells. The cell concentration was adjusted to $1x10^7$ cells/ml. Flow cytometry antibodies were added according to the manufacturer's instructions (Anti-mouse CD45, Biolegend, 103129; Anti-mouse CD3, Biolegend, 100215; Anti-mouse CD4, Biolegend, 100525; Anti-mouse CD8a, Biolegend, 100705; Anti-mouse CD11b, Biolegend, 101211; Anti-mouse FoxP3, Biolegend, 126419; Anti-mouseF4/80, Biolegend, 123133; Anti-mouse CD80, Biolegend, 104707; Anti-mouse CD206, Biolegend, 141723). The cells were resuspended in 4% paraformaldehyde and fixed at room temperature for 20 minutes. For intracellular cytokine staining, 1x permeabilization buffer was added to permeabilize the cells. The prepared single-cell suspension was analyzed using a flow cytometer (Beckman Coulter, USA), and the data was analyzed using FlowJo software (Treestar).

Immunohistochemistry

The immunohistochemical staining process involved fixation, dehydration, wax embedding, sectioning, dewaxing, hydration, antigen retrieval, serum blocking, primary and secondary antibody incubations, chromogenic development, counterstaining, dehydration, and mounting. Subsequently, slides were analyzed under an inverted microscope to assess the expression of different markers at 3 or 9 days post-radiofrequency ablation, with semi-quantitative analysis of positive cells conducted in 5 random fields per slide.

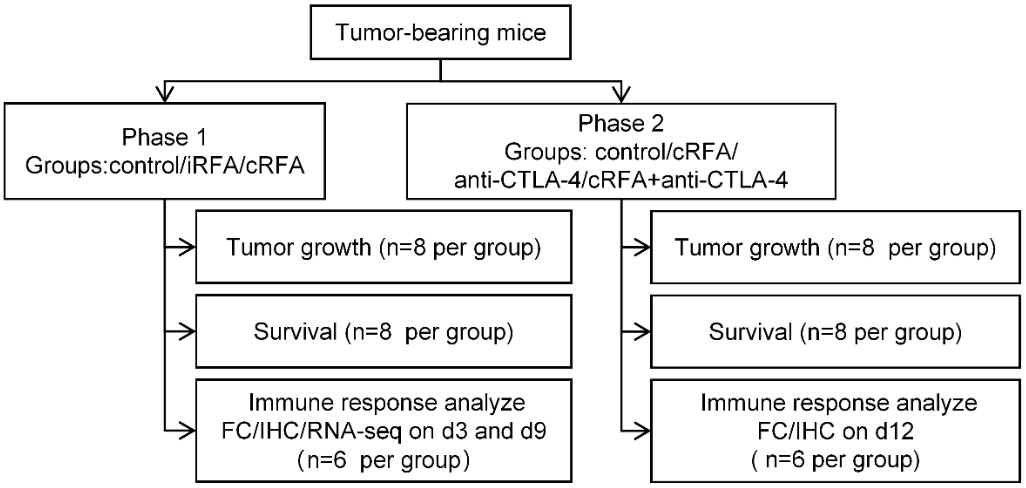

Figure S1. Study design flow chart CONSORT-style flowchart detailing animal allocation, experimental cohorts, and analysis pathways.

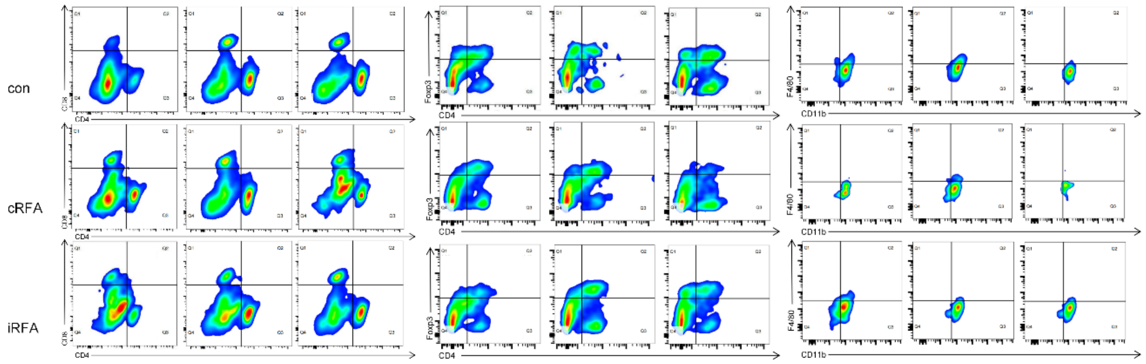

Figure S2. Individual results from flow cytometry analysis show the populations of CD8^{+} T cells, Tregs, and TAMs on Day 3.

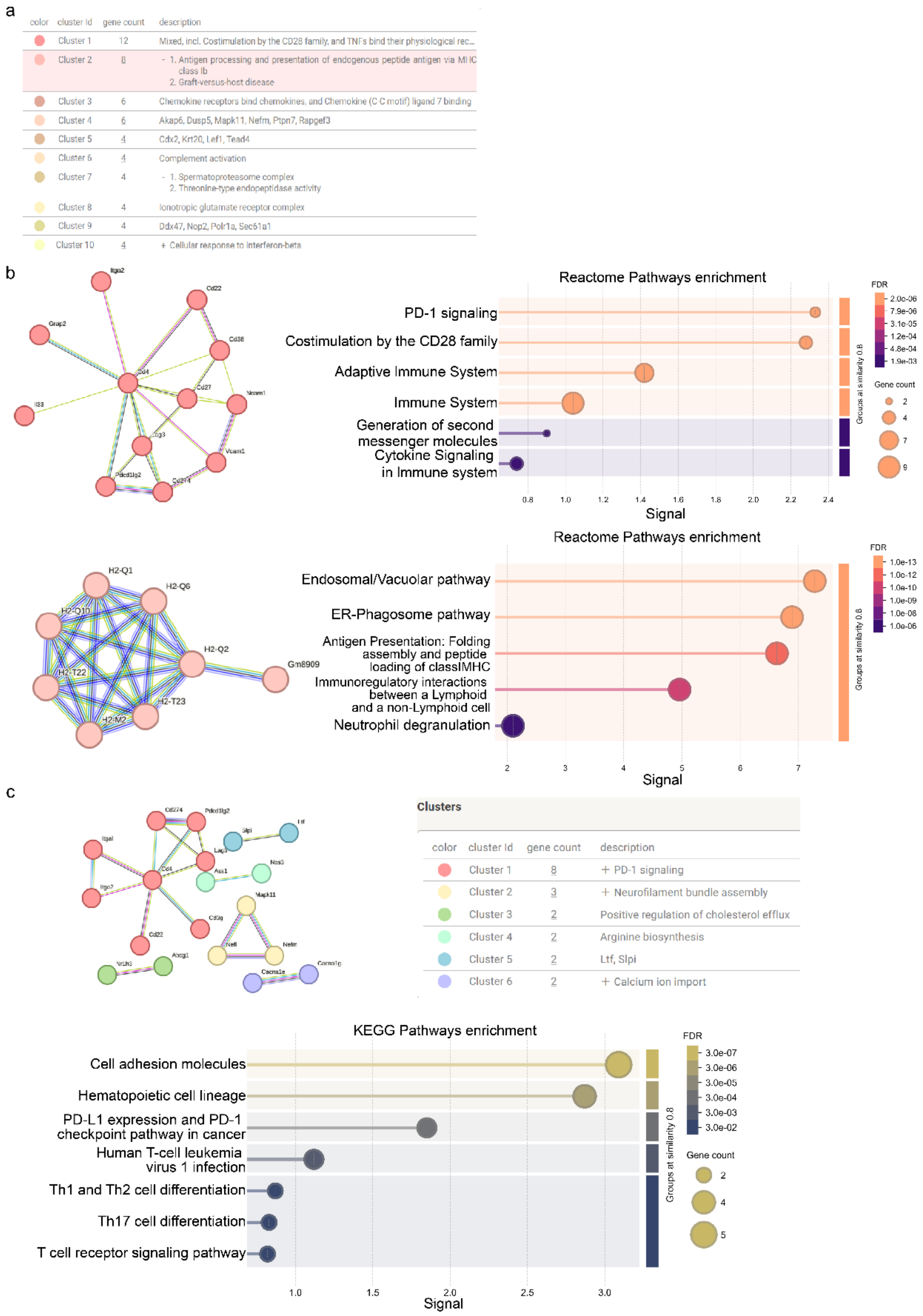
a
color cluster Id gene count description
Cluster 1 12 Mixed, incl. Costimulation by the CD28 family, and TNFs bind their physiological rec...
Cluster 2 8 1. Antigen processing and presentation of endogenous peptide antigen via MHC class Ib
2. Graft-versus-host disease
Cluster 3 6 Chemokine receptors bind chemokines, and Chemokine (C-C motif) ligand 7 binding
Cluster 4 6 Akap6, Dusp5, Mapk11, Nefm, Ptpn7, Rapgef3
Cluster 5 4 Cdx2, Krt20, Lef1, Tead4
Cluster 6 4 Complement activation
Cluster 7 4 1. Spermatoproteasome complex
2. Threonine-type endopeptidase activity
Cluster 8 4 Ionotropic glutamate receptor complex
Cluster 9 4 Ddx47, Nop2, Polr1a, Sec61a1
Cluster 10 4 + Cellular response to interferon-beta
b
Reactome Pathways enrichment
PD-1 signaling
Costimulation by the CD28 family
Adaptive Immune System
Immune System
Generation of second messenger molecules
Cytokine Signaling in Immune system
Signal
FDR
Gene count
Groups at similarity 0.8
Reactome Pathways enrichment
Endosomal/Vacuolar pathway
ER-Phagosome pathway
Antigen Presentation: Folding assembly and peptide loading of classIMHC
Immunoregulatory interactions between a Lymphoid and a non-Lymphoid cell
Neutrophil degranulation
Signal
FDR
Groups at similarity 0.8
H2-Q1
H2-Q6
H2-D10
H2-Q2
Gm8909
H2-T22
H2-T23
H2-M2
c
Clusters
color cluster Id gene count description
Cluster 1 8 + PD-1 signaling
Cluster 2 3 + Neurofilament bundle assembly
Cluster 3 2 Positive regulation of cholesterol efflux
Cluster 4 2 Arginine biosynthesis
Cluster 5 2 Ltf, Slpi
Cluster 6 2 + Calcium ion import
KEGG Pathways enrichment
Cell adhesion molecules
Hematopoietic cell lineage
PD-L1 expression and PD-1 checkpoint pathway in cancer
Human T-cell leukemia virus 1 infection
Th1 and Th2 cell differentiation
Th17 cell differentiation
T cell receptor signaling pathway
Signal
FDR
Gene count
Groups at similarity 0.8

Figure S3. Genes associated with immune system process were induced 3 days after RFA. (a) The MCL clusters of genes after complete RFA compared with the control group. (b) The Reactome pathway analysis of up-regulated genes in the top 2 clusters showed that immune system and PD-1 signaling were involved. (c) The PPI network and the KEGG pathway analysis of the genes in cluster1 described as PD-1 signaling demonstrates down-regulated immune functions at 72 hours after iRFA.

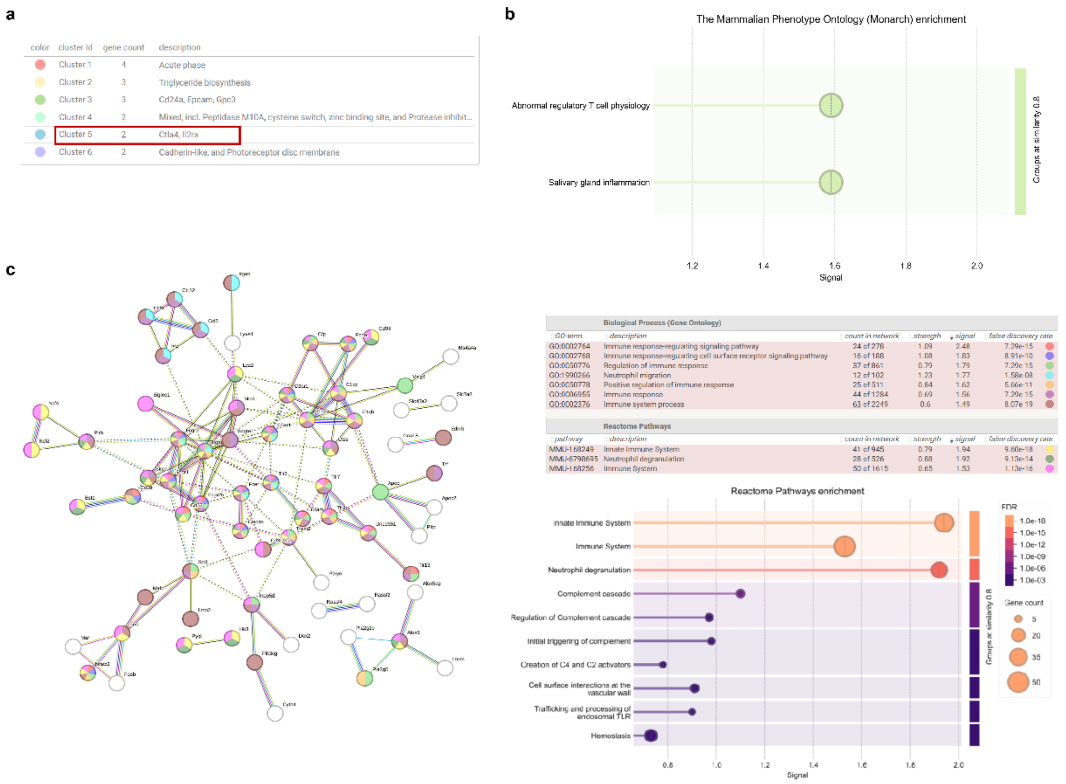

Figure S4. Genes associated with immune system process were induced 9 days after RFA. (a) The MCL clusters of genes at 9 days after complete RFA compared with the control group showed demonstrates that CTLA-4 was up-regulated. (b) MPO enrichment analysis of genes after complete RFA compared with the control group. (c) PPI plot and Reactome Pathways enrichment analysis of 60 genes down-regulated demonstrates multiple down regulated immune functions after incomplete RFA.

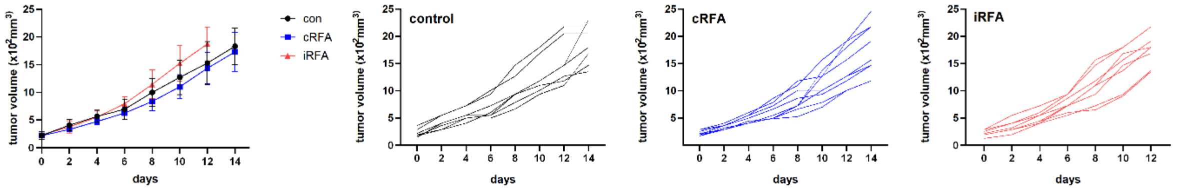

Figure S5. Tumor growth trajectories with 95% confidence interval bands and individual per-mouse tumor growth of phase 1.

Eur Radiol Exp (2026) Zhao K, Shen Y, Yang W, et al.
